# Development and Validation of a Novel Tool to Measure Medication Adherence for Noncommunicable Diseases in India: Protocol for an Exploratory Sequential Mixed Methods Multicentric Study

**DOI:** 10.2196/60805

**Published:** 2024-12-03

**Authors:** Joe Thomas, Maria Jose, Priyanka Rajmohan, Farah Naaz Fathima, Hisham Moosan, Nisha K Jose, Thekkumkara Surendran Anish, Mohan Bairwa, Tulika Goswami Mahanta, Aditi Apte, Jerin Jose Cherian, Praveenlal Kuttichira, Ravi Prasad Varma P

**Affiliations:** 1 Department of Community Medicine Jubilee Mission Medical College and Research Institute Thrissur India; 2 Department of Pharmacology Jubilee Mission Medical College and Research Institute Thrissur India; 3 Department of Community Medicine St Johns Medical College Bengaluru India; 4 National Institute for Implementation Research on Non-Communicable Diseases Jodhpur India; 5 Indian Council of Medical Research New Delhi India; 6 Department of Community Medicine Government Medical College Manjeri India; 7 Centre for Community Medicine All India Institute of Medical Sciences New Delhi India; 8 Department of Community Medicine Assam Medical College Dibrugarh India; 9 King Edward Memorial Hospital Research Centre Pune India; 10 Jubilee Mission Medical College and Research Institute Thrissur India; 11 Achutha Menon Centre for Health Science Studies Sree Chitra Tirunal Institute for Medical Sciences and Technology Trivandrum India

**Keywords:** medication adherence, noncommunicable diseases, self-report, psychometric properties, validation, reproducibility of results

## Abstract

**Background:**

In high-income countries, only 50% of patients treated for chronic diseases adhere to the prescribed treatment. This issue is even more pronounced in resource-limited countries. Medication adherence scales are simple, low-cost approaches to identify nonadherence in clinical practice. In India, nonadherence to medication varies from 18.7% to 74%, assessed using scales validated in the Western population, as there is no validated medication adherence tool contextualized to the Indian setting. The phrasing of questions in scales validated elsewhere and its interpretations may vary when applied in Indian patients unless accounting for the unique cultural, social, and economic factors influencing medication adherence in India. This could result in inaccurate reports of adherence behavior.

**Objective:**

This study aims to develop and validate a novel medication adherence tool for select noncommunicable diseases (diabetes mellitus, hypertension, chronic obstructive pulmonary disease, bronchial asthma, and coronary artery disease) in the Indian population.

**Methods:**

An exploratory sequential mixed methods design will be used, beginning with a qualitative phase where the construct of the scale is defined and preliminary items are generated through a scoping review, focus group discussions, and in-depth interviews. This will be followed by the tool’s development phase, including an expert panel review and item revision. Finally, a quantitative phase in 4 zones in India (North, South, East, and West) will be conducted to confirm and validate the newly developed scale.

**Results:**

In the first phase, we will frame the construct definition and develop an inventory of potential items for the proposed medication adherence tool. In the second phase, item-level and scale-level content validity indices, along with content validity ratio, will be estimated. In the third phase, we will conduct an item reduction analysis and determine the scoring matrix and item weightage after expert review. We will assess the tool's psychometric properties, plot the receiver operating characteristic (ROC) curve to set an adherence cut-off score, and compute the construct validity and test-retest reliability from the quantitative survey.

**Conclusions:**

A medication adherence tool for noncommunicable diseases, developed after ensuring it is ethnically, culturally, and linguistically appropriate incorporating stakeholder perspectives and validated in community settings, would offer a real-world perspective of adherence. The tool will have 2 versions for clinical practice and research, aiding policy makers in adopting tailored adherence policies.

**International Registered Report Identifier (IRRID):**

PRR1-10.2196/60805

## Introduction

India is undergoing an epidemiological health transition with an increasing burden of noncommunicable diseases (NCDs). NCDs contribute to approximately 5.87 million deaths, accounting for 60% of all deaths in India. This represents two-thirds of the total deaths caused by NCDs in the World Health Organization’s South-East Asia Region [1]. In high-income countries, only 50% of patients treated for chronic diseases adhere to the prescribed treatment. This issue is even more pronounced in resource-limited countries [1]. The key contributor to the increasing burden of morbidity and mortality due to NCDs is patients’ poor control status related to medication nonadherence [2]. Diabetes, hypertension, and bronchial asthma rank among the highest diseases reporting medication nonadherence [3,4]. Improving medication adherence requires assessment using a valid and reliable adherence scale, considering the various dimensions of adherence. Objective measures include pill counts, electronic monitoring, secondary database analysis, and biochemical measures, which are often impractical and resource-intensive. Subjective measures like self-reports and health care professional assessments rely on data, which could be influenced by social desirability and recall biases and might not capture complex cultural and socioeconomic factors. Nevertheless, they are cost-effective, nonintrusive, user-friendly, easy to administer, and can capture patients’ concerns, helping individualize adherence interventions.

Globally, the psychometric properties of different medication adherence scales have demonstrated heterogeneous results when applied in different settings and disease conditions. Studies by Sakthong et al [5] and Zongo et al [6] reported that the Morisky Medication Adherence Scale (MMAS) demonstrated sensitivity as low as 51% and low reliability values in patients with type 2 diabetes mellitus (T2DM). The 5-item brief version of the Medication Adherence Report Scale (MARS-5) was found to be less sensitive in measuring medication adherence to asthma and other chronic disease conditions [7,8].

In India, nonadherence to medication varies from 18.7% to 74%, [2,9-11], which is assessed using scales validated in the Western population. Currently, we do not have a validated medication adherence tool contextualized to the Indian setting. The phrasing of questions in scales validated elsewhere and its interpretations may vary when applied to Indian patients unless accounted for unique cultural, social, and economic factors influencing medication adherence in India. This could result in inaccurate reports of adherence behavior.

The most commonly used adherence scales in these studies are the MMAS-4 and MMAS-8, Self-Efficacy for Appropriate Medication Use Scale, Beliefs about Medicines Questionnaire (BMQ), MARS, MARS-5, Hill Bone Compliance Scale, Adherence to Refills and Medications Scale (ARMS), and General Medication Adherence Scale [12-20]. However, these adherence scales have their own strengths and limitations. MMAS-8 has been extensively validated for a range of disease conditions. However, cost-related nonadherence and health system–related dimensions have not been explored in MMAS. Moreover, mandatory license fees preclude its widespread use in resource-limited settings. Scoring ambiguity (dichotomous response) can result in lower reliability estimates. The absence of validation in patients with low literacy levels (MMAS, BMQ, and MARS) is another factor that can affect the usability of these scales in resource-limited nations. Furthermore, few studies have considered the psychometric properties of adherence scales in the Indian context [21,22]. Concurrent validity against an objective measure of adherence has been assessed in only a few scales (BMQ).

Moreover, the linguistic validity of these translated versions of the tools has not been assessed by cognitive piloting considering the patient’s perspectives and the acceptability of the tool. Measuring self-efficacy is an important factor in patient enablement to motivate and monitor their adherence behavior based on the scoring. However, this dimension of adherence behavior has not been explored in scales such as MMAS, MARS, and ARMS. Furthermore, India has mixed health care systems, where people’s access to and use of medicines are dependent on their socioeconomic, cultural, and religious backgrounds. Out-of-pocket expenditure is an important area of health economics, especially in resource-limited countries where most patients pay direct medical costs [23,24]. Thus, the existing tools lack this important domain for measuring adherence. We aim to develop a novel indigenous medication adherence tool for select NCDs that is ethnically, culturally, and linguistically appropriate. The tool’s various constructs will be developed after exploring expert opinions and patients' perspectives on adherence behavior. To ensure that the contents are relevant to the local population, the tool will be linguistically validated across different geographical zones in India. Unlike other medication adherence scales that have been validated in hospital settings, where patients are likely to be more adherent, we propose validating our tool in community settings, which would give a more accurate real-world perspective on adherence. This tool, which is envisaged to be developed in 2 versions, could be used across chronic disease conditions both in routine clinical practice (short version) and research settings (long version). This can help policy makers understand and identify reasons for nonadherence and then consequently adopt tailored policies to improve adherence. Thus, this study aims to develop a medication adherence tool and evaluate its psychometric properties to measure adherence for select NCDs in the Indian population. Furthermore, it aims to describe stakeholder perceptions of medication adherence through qualitative research.

## Methods

### Overview of the Study Design and Setting

The study will use an exploratory sequential mixed methods approach involving qualitative and quantitative components. This approach is widely recognized for its scale construct development and validation [25]. The study design includes three phases: (1) a qualitative phase defining the construct of the instrument; (2) an instrument development phase including item generation and revision; and (3) a confirming quantitative phase to test the instrument. In the qualitative phase, along with a scoping review, focus group discussions (FGDs), in-depth interviews (IDIs), and key informant interviews (KIIs) will be conducted among stakeholders to explore the scale construct and collect detailed information. The findings of the qualitative study will be systematically coded, and themes will be generated. These themes will be converted to scale items. In the instrument development phase, the resulting set of questions will be reviewed by a multidisciplinary team of experts. The items will then be reviewed for clarity and comprehension, and appropriate revisions will be made. After this process, the quantitative phase will begin, which will be conducted in 2 stages. A preliminary survey will be conducted to administer the new instrument among a sample of the target population with select NCDs. Item responses will be subjected to factor analysis to collect evidence for construct validity. Exploratory factor analysis with principal axis factor analysis will be used to retrieve the factors, reduce the items, and examine the factor structure. The strength of the factor loadings will indicate which items are the best, good, or acceptable on the scale.

In the second stage, the scale will undergo validation across 4 zones of India (North, South, East, and West). Our scale is designed to measure medication adherence among NCD patients in India, taking into account the significant cultural, social, and linguistic differences across regions. Scale evaluation will be carried out by examining the individual item and domain score to identify any floor and ceiling effect. Furthermore, concurrent validity against a gold standard will be estimated.

### Study Procedure

#### Phase 1 (Qualitative): Defining the Construct and Generating the Item Pool

Initially, we will conduct a scoping review of the existing literature on medication adherence scales for NCDs validated in the adult population. This review of current global and Indian scenarios will help us methodically compile and analyze the evidence derived from the different medication adherence scales currently in use for NCDs. This will be done by considering their psychometric characteristics, including the delineation of quality benchmarks such as sensitivity, specificity, convergent validity, and reliability metrics. Furthermore, we will synthesize evidence on various medication adherence scales to identify their limitations, establish a working definition of medication adherence, determine the dimensionality of its constructs, and develop a list of potential items for creating a medication adherence scale tailored to the Indian context.

This process will be followed by FGDs, IDIs, and KIIs conducted across health centers in Kerala. Incorporating input from the target population when creating the questionnaire items will ensure that the resulting questionnaire accurately captures their perspectives. This process facilitates the inclusion of items that are acceptable, comprehensive, and relevant to the disease condition [26].

For FGDs, the participants will include staff members from primary health centers (PHCs) who have at least 2 years of experience managing NCD patients, including medical officers, staff nurses, pharmacists, health inspectors, public health nurses, and accredited social health activist workers. The selection of PHCs will be based on permission obtained from the health authorities and logistical considerations. The procedure for conducting FGDs is detailed in the FGD guide, provided in Multimedia Appendix 1. For IDIs, the participants will include patients with NCDs and their caregivers (1 adult and 1 older adult >60 years of age per disease condition). Participants will be recruited using convenience sampling. KIIs will include consultants specializing in NCDs from both government and private sectors, general practitioners, and NCD program officers. Details of the interview procedures are provided in the IDI and KII guides, included in Multimedia Appendices 2 and 3.

Data collected from these interviews and FGDs will undergo thematic analysis and triangulation to facilitate a more comprehensive understanding. Braun and Clarke's 6-step framework, including familiarization with data, generating initial codes, searching for themes, reviewing themes, defining and naming themes, and producing the report, will be used in our thematic analysis [27]. We will introduce triangulation by using multiple data sources and peer debriefing sessions and will consider member-checking where feasible. To ensure repeatability, we will document each stage of the analysis process to ensure minimal researcher bias. For confirmability, the findings will be supported by representative quotes from participants. The study context and participant characteristics will be comprehensively described to ensure generalizability.

**Figure 1:**
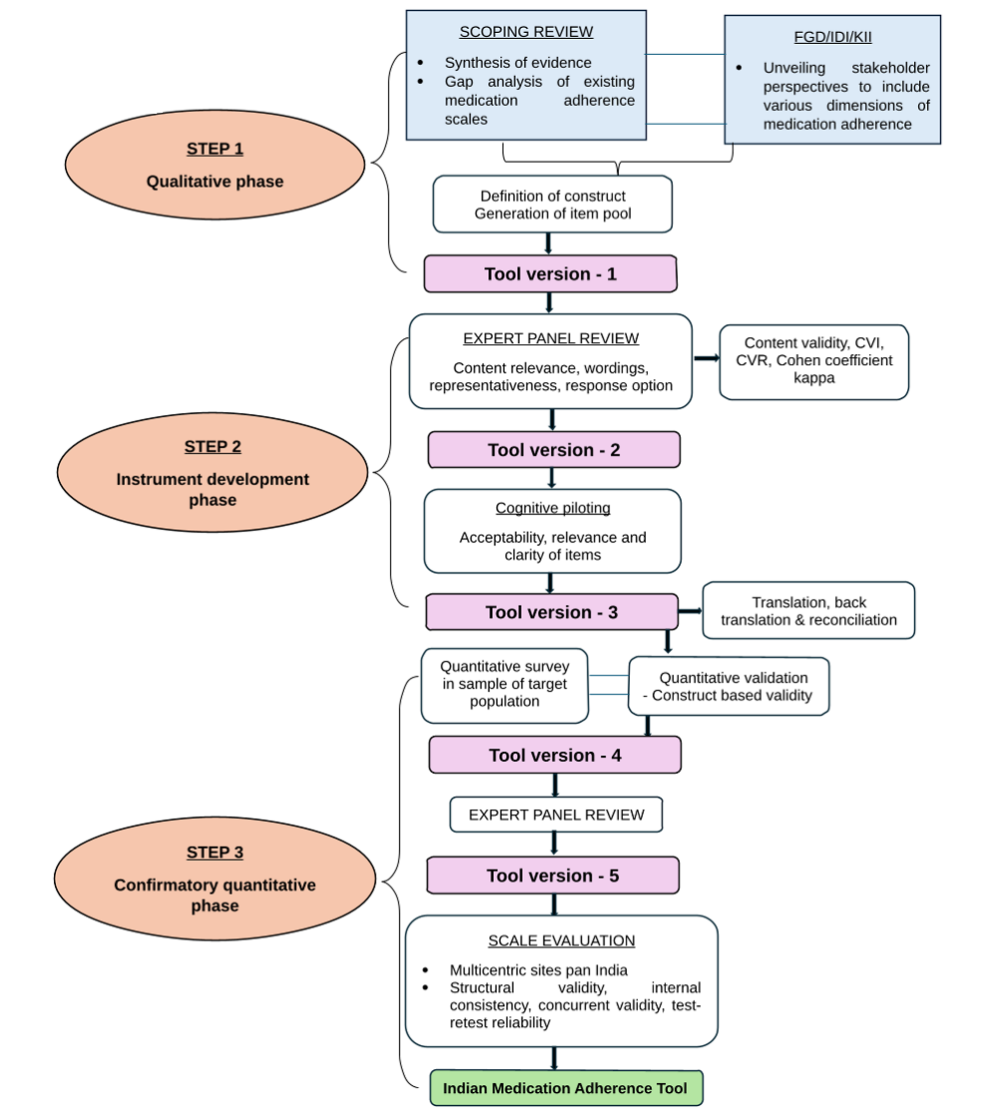
Study procedure and data collection. CVI: content validity index; CVR: content validity ratio.

This process will enable us to determine which items to add, delete, or modify from the initial questionnaire and identify potential additional domains of medication adherence for inclusion in the assessment tool. The outcome of this whole process will be the development of version 1 of the adherence tool. Figure 1 outlines the study procedure and data collection.

#### Phase 2: Instrument Development Phase

In this phase, evidence on the content-based validity of the proposed scale will be estimated to determine whether the items comprehensively represent the scale construct to be measured. This will be done by a panel of experts, whereby each item constituting the domain will be evaluated for content relevance, wording, representativeness, and response options. The expert panel will consist of 10 to 12 professionals from various disciplines, including physicians, cardiologists, pulmonologists, endocrinologists, general practitioners, and pharmacists. All panel members will be experienced in the construct of medication adherence and will be selected from across the 4 zones of India (North, South, East, and West).

Subsequently, the assessments will be quantified using formalized scaling and statistical procedures, including content validity ratio, content validity index, and Cohen coefficient kappa. Each item will either be accepted, rejected, or modified based on the results. This process will result in version 2 of the proposed tool.

The cognitive piloting (pretesting) of tool version 2 will be done in 2 rounds, with 1 participant from each disease condition under study in each round.

Cognitive piloting will ensure that the questionnaire assesses the acceptability, relevance, and clarity of the items. Cognitive piloting ensures that the questions are appropriately phrased, cover relevant areas, and maintain a logical flow. This step will also verify that questions are well-worded, understandable, and acceptable to participants before the main data collection [28]. Pretesting of the questionnaire will be conducted with participants who are not included in the study. Feedback from participants will be collected, and the questions will be rejected, amended, clarified, or augmented where necessary. This will comprise version 3 of the draft item tool. The translated version will undergo back translation and reconciliation before proceeding to the quantitative phase.

#### Phase 3 (Quantitative): Administering the Scale for Quantitative Validation in the Target Population

A quantitative survey will be conducted among a sample of the target population in the South zone, which serves as the field practice area for the lead site.

##### Inclusion Criteria

Participants will be individuals over 18 years of age undergoing drug treatment for at least 1 year for any of the select NCDs, including T2DM (oral antidiabetic drugs), hypertension, coronary artery disease (CAD), chronic obstructive pulmonary disease (COPD), and bronchial asthma.

##### Exclusion Criteria

Individuals with cognitive or psychological disorders, or debilitating illnesses that could impair their ability to respond will be excluded from the study.

##### Item Analysis and Expert Panel Review

Item responses will be analyzed via a series of statistical analyses for evidence of construct-based validity, including internal reliability (Cronbach alpha) and item correlations. Principal Axis Factor Analysis with oblique rotation will be used for item reduction, as this analysis allows for the removal of error variances and the correlation of extracted factors. The item reduction analysis will ensure that only parsimonious, functional, and internally consistent items are retained. Following this step, version 4 of the draft item tool will be finalized.

After item reduction, a second round of expert panel review (Delphi) will take place. The tool will be presented to a panel of experts to reach a consensus on the developed items and domains under which each item is categorized in the tool. Experts will also help with naming the domains and addressing any cross-loading factors on the same item, if applicable. This process will result in version 5 of the tool.

##### Scale Evaluation

The final version of the questionnaire (version 5) will be administered to a large representative sample of respondents from different zones across India to field test the tool’s content, usability, and appropriateness for the target population. The study centers will be selected from the 4 zones across India. We will select centers that have both urban- and rural-attached facilities. Participants will be recruited purposively from these zones to ensure heterogeneity in socioeconomic, geographic, and cultural characteristics. The inclusion and exclusion criteria will be the same as those of the initial survey.

The final questionnaire will be translated into 4 different languages (Malayalam, Hindi, Assamese, and Marathi), followed by back-translation and reconciliation. The structural validity and internal consistency of the tool will be established at this stage. Individual item and domain scores will be examined for floor and ceiling effects. Sociodemographic details and medication adherence scores will be collected using the version 5 tool. The direct pill count will be assessed by a trained health worker after 2 consecutive visits at an interval of 30 days. In a subset of 10% (n=30) of participants from each site, we plan to provide a medication event monitoring system (MEMS) during the enrollment. We will then revisit these participants after 30 days to calculate the pill count. Wilcoxon signed-rank tests and Bland-Altmann analysis will be performed for each item, domain, and overall scale. Intraclass coefficients will be computed for domains and overall scale. This will establish the concurrent validity, sensitivity, and specificity of the new tool against the MEMS. If a participant is taking multiple medications, their pill-taking behavior will be assessed for only 1 medication. The scale will be readministered after 30 days for this subset in each zone to assess test-retest reliability. The criterion validity for the sample with hypertension will be estimated as a measure of blood pressure control. This will involve using the mean of 2 blood pressure readings taken 5 minutes apart.

##### Sample Size

For the qualitative phase, we will conduct between 4 and 5 rounds of FGDs until theoretical data saturation occurs. For IDIs, a total of 8 patients and 8 caregivers will be interviewed. For the KIIs, 3 treating physicians (1 consultant each from a private and a government sector, 1 general practitioner) and 1 district NCD program officer will be interviewed.

In the quantitative phase, the preliminary survey will be conducted among approximately 300 participants (between 8 and 10 respondents for each item). For the cross-sectional study across the 4 zones, for scale evaluation, the sample size was calculated based on a sensitivity of 0.81, with an absolute precision of 5% and a 95% CI. The total estimated sample size is 1200 [12]. We plan to recruit 300 participants from each of the 4 study sites located in different zones across India. This will be at a recruitment rate of 75 participants per disease condition studied per site.

##### Data Management Plan

The data will be collected using structured case record forms (CRFs), which will be pilot-tested and validated. Relevant data will be captured on electronic CRFs and transmitted via the internet to the lead site. We will verify the data for appropriateness, run quality checks, and generate queries for clarification. Interviewers will be trained in data collection techniques, and interview times will be automatically logged. Regular supervisory visits and quality control protocols will be implemented. A quality assurance (QA) manager will monitor project activities, while the principal investigator and team will randomly check participant charts. The QA manager will independently perform source data verification, validate field visits, and review field diaries to assess data collection methods.

For the qualitative study, a descriptive content analysis of the transcripts will be carried out and subthemes will be generated. Thematic analysis and interpretive description will be done. NVivo software (version 12; QSR International) will be used to further analyze the qualitative data. In terms of content validity, the relevance of questions and ease of administration will be assessed and analyzed using item-level and scale-level content validity indices, as well as the content validity ratio, calculated with modified kappa statistics.

In the quantitative study, data will be entered into Microsoft Excel (IBM Corp) and analyzed using SPSS software (version 25; IBM Corp) for the tool validation and cross-sectional study. Item reduction will be carried out using exploratory factor analysis, including correlation matrix diagnostics, communalities, factor extraction based on eigenvalues, and factor identification based on factor loadings. Correlation matrices, the Kaiser-Meyer-Olkin test, the Bartlett test for sphericity, and determinant and individual measures of sampling adequacy will be used for establishing item adequacy for factor extraction and rotation. Cut-offs of item communalities will be determined iteratively, starting at 0.5 and, if necessary, reducing to no less than 0.3. The number of factors to retain will be identified using eigenvalues and scree plots, with factor loadings for individual items obtained from the pattern matrix. Wilcoxon signed-rank tests and Bland-Altmann analysis will be conducted for each item, domain, and overall scale. Intraclass coefficients will be computed for the domains and the overall scale. Adherence scores will be calculated as proportions using the validated tool.

### Ethical Considerations

The study will be conducted after obtaining approval from the institutional ethics committee (reference no 64/23/IEC/JMMC&RI). All participants will be informed about the study’s purpose and assured that participation is entirely voluntary. They will be told that refusing to participate will have no consequences and that they may withdraw at any time without providing an explanation. No incentives, other than contributing to scientific knowledge, will offered to encourage participation.

Participants will be given an information sheet summarizing key information about the study and data protection measurements. Written informed consent will be obtained from all participants, with privacy and confidentiality assured. The consent forms are attached in Multimedia Appendix 4. The consent process and participant protection measures will be documented following institutional and nationally mandated guidelines.

All information, including participant names, will be kept confidential. Only the principal investigator and selected project staff will access the data. Relevant data at the collaborator site will be captured on electronic CRFs using REDCap (Research Electronic Data Capture) and transmitted securely to the lead site. Hard copies of paper forms and documents will be stored securely under lock and key, while electronic data files will be downloaded and stored as password-protected files. Anonymized data files will be uploaded to cloud storage to ensure data privacy and confidentiality, protecting sensitive information from unauthorized access, misuse, or breaches. For this study’s qualitative component, recordings of interviews and focused group discussions will be stored in secure folders with restricted access. Standard Good Clinical Practice (GCP) will be followed to ensure accurate, reliable, and consistent data collection.

## Results

We will compute the item-level and scale-level content validity indices and content validity ratios to evaluate the validity of each item. Items will be then classified as rejected, accepted, or modified in phase 2 of the study. In phase 3, we will compute the Cronbach alpha and assess the contribution of each item to the extracted factors. Items that decrease alpha or do not contribute meaningfully will be scrutinized, with decisions made based on theoretical appropriateness, content validity ratios, and communality. Following an expert panel review, we will establish a scoring matrix and assign weightings to each item. This evaluation will lead to further adjustments, resulting in a final selection of items that will be accepted, modified, or rejected based on the review process. Furthermore, for the purpose of use in routine clinical settings, we will decide on 2 best fit global questions based on the consensus of the expert panel and content validity results. Construct validity for the sample with hypertension will be estimated as a measure of blood pressure control (mean of the 2 blood pressure values taken 5 minutes apart during the first visit). The scale will be readministered after 30 days for this subset in each zone to assess test-retest reliability.

The primary end point of the study will be the psychometric properties of the tool, including sensitivity, specificity, content validity index, content validity ratio, Cronbach alpha, and factor structure. Additionally, the receiver operating characteristic (ROC) curve will be used for determining cut-off score between adherent and nonadherent participants. The secondary end points will include the determinants of medication adherence from the perspectives of providers (doctors, pharmacists, nurses, and program officers) and end users (patients), as well as prevalence estimates of adherence for the conditions studied (T2DM, hypertension, CAD, COPD, and bronchial asthma).

The total duration of the study project is 36 months. In the first year, by February 3, 2024, evidence was synthesized from the existing literature. Focus groups and in-depth interviews for item generation for the proposed medication adherence tool were conducted between May 3, 2024, and July 3, 2024. In the second year, we will conduct an expert panel review and cognitive piloting by February 2, 2025, and the tool developed will be used for survey administration by April 2, 2025. The items will be further modified based on item reduction analysis by June 2, 2025, followed by a second round of expert panel review by August 2, 2025. For the final validation of the proposed medication adherence tool, we will conduct a cross-sectional study across the 4 zones across India between October 3, 2025, and April 2, 2026. We expect to publish our scoping review by November 2024, the stakeholder perspectives by September 2025, and the validation of the medication adherence tool at the end of year 3, by October 2026.

## Discussion

### Expected Findings

Currently, we do not have a validated medication adherence tool contextualized to the Indian setting. In low-resource settings, existing medication adherence scales should be used with caution due to the following reasons. First, most of these questionnaire scales were originally validated in Western contexts and may not be directly applicable in diverse settings like India. These scales should be assessed for cross-cultural equivalence, considering the varied linguistic, socioeconomic, and cultural extremes of literacy prevalent in India. The phrasing of questions in scales validated elsewhere and its interpretations may vary when applied among Indian patients. This will result in mixed findings on accuracy, reliability, and validity in patients with various chronic disease conditions.

Only a few studies have examined the psychometric properties of adherence scales such as MMAS, MARS, BMQ, and ARMS [12,13]. In Thailand [5], among patients with T2DM, MMAS-8 reported low sensitivity and predictive accuracy (51% and 43%, respectively), with a moderate specificity (64%). A study on the psychometric evaluation of MARS in caregivers of low-income, urban, African American children with poorly controlled asthma, conducted by Margolis et al [7], showed marginal fit in the Confirmatory Factor Analysis and a lack of associations with objective measures.

Scribano et al [29] reported that the Italian version of the MARS showed satisfactory internal consistency and test-retest reliability but showed low convergent validity. Furthermore, MARS has not been validated for use in patients with low literacy and does not assess self-efficacy. The cost factor, out-of-pocket expenditure, and patient self-efficacy dimensions of medication adherence were not considered in the ARMS. Additionally, the sensitivity and specificity of ARMS and the Self-Efficacy for Appropriate Medication Use Scale have not been reported and compared to clinical outcomes [14,18].

To address the limitations of existing medication adherence scales, we intend to examine the convergent validity of the new tool by comparing it with objective measures such as direct pill count and MEMS. Our novel, indigenously developed tool will include dimensions such as patient medication-taking behavior, comorbidity, pill burden, and aspects measuring self-efficacy. The proposed tool will be suitable for patients across all literacy levels. To accommodate patients with low literacy, some items will include pictograms to facilitate understanding and responses.

Furthermore, India has mixed health care systems, where people’s access to and use of medicines are influenced by their socioeconomic status. Out-of-pocket expenditure is an important area of health economics, especially in resource-limited countries where most patients pay direct medical costs. Existing tools lack a consideration for this important domain when measuring adherence. Hence, the various characteristics of the proposed tool would be developed after considering expert opinions and patients' perspectives on adherence behavior. This will help us to assess various dimensions of adherence. The cost factor and out-of-pocket expenditure on medications will also be included as a dimension in our tool. To ensure that the contents are relevant to the local population, our tool will be linguistically validated across different geographical zones in India. Validation in community settings, as opposed to hospital settings, conducted in other preexisting tools, will provide real-world evidence on adherence. Moreover, we intend to develop a shorter version of the medication adherence tool, which could be integrated into routine clinical practice. Between 2 and 3 best-fit global questions in this short version will be selected after considering the content validity indices, expert consensus, and communality. This is a simple and rapid method for assessing medication adherence in point-of-care or clinical settings.

We acknowledge the potential limitations of our study and have outlined strategies to mitigate them. First, because our study will be conducted with specific populations, the findings may have limited generalizability and may not fully represent diverse population characteristics. To address this limitation, we will provide recommendations for future studies to test the findings in different contexts. Second, since our study relies on interviews/self-reported data, there may be potential recall bias or social desirability bias. To minimize these effects, we will use standardized, open-ended questions and ensure participant confidentiality. Third, researcher perspectives might affect the qualitative portion of the study. We plan to address this through reflexive data collection, analysis methods, and team discussions [30]. Finally, the timing of data collection might influence the findings, particularly if impacted by including seasonal factors or significant social events. We will consider these contextual influences while interpreting the results of our study.

### Conclusion

A medication adherence tool for selected NCDs, developed to be ethnically, culturally, and linguistically appropriate through expert opinion and patient perspectives and validated in community settings, will provide a real-world perspective on adherence. The tool will have two versions for clinical practice and research, aiding policy makers in adopting tailored adherence policies.

## Appendix

Multimedia Appendix 1 Focus group discussion guide.

Multimedia Appendix 2 In-depth interview guide for patients.

Multimedia Appendix 3 In-depth interview guide for caregivers.

Multimedia Appendix 4 Consent forms.

Multimedia Appendix 5 Peer-review report by the National Task Force (NTF) on Safe and Rational use of Medicines (SRUM) - Indian Council of Medical Research (ICMR) Central Coordinating Unit (CCU) (New Delhi, India).

## Abbreviations

ARMS: Adherence to Refills and Medications Scale
BMQ: Beliefs about Medicines Questionnaire
CAD: coronary artery disease
COPD: chronic obstructive pulmonary disease
CRF: case record form
FGD: focus group discussion
GCP: Good Clinical Practice
IDI: in-depth interview
KII: key informant interview
MARS: Medication Adherence Report Scale
MEMS: medication event monitoring system
MMAS: Morisky Medication Adherence Scale
NCD: noncommunicable disease
PHC: primary health center
QA: quality assurance
REDCap: Research Electronic Data Capture
ROC: receiver operating characteristic
T2DM: type 2 diabetes mellitus

## Data Availability

The data sets generated during and /or analyzed during the study are available from the corresponding author upon reasonable request.
