# Supplementary material for: Development and Validation of a Novel Tool to Measure Medication Adherence for Noncommunicable Diseases in India: Protocol for an Exploratory Sequential Mixed Methods Multicentric Study
Source: JMIR Res Protoc. 2024 Dec 3;13:e60805. doi: 10.2196/60805 (PMC11653035; doi:10.2196/60805)
Supplement: Multimedia Appendix 1 [file resprot_v13i1e60805_app1.docx]

**FOCUS GROUP DISCUSSION GUIDE**

**Introduction**

1. **Introduction of facilitator/moderator and note taker**

"Welcome and thank you for coming today. My name is _________ and these are my colleagues _____________ and __________________. This focus group is being carried out for an ICMR Project intended to develop a tool to assess the medication adherence among patients with T2 DM on OHA, hypertension, CAD or COPD/Bronchial Asthma . I am [Identify yourself and the other team members – with the role in the project .] We are conducting this focus group to explore the viewpoints of health care personnel who have direct contact and interaction with patients suffering from Type II Diabetes Mellitus, Hypertension, asthma/COPD or Coronary Artery Diseases . From the discussion, we hope we will be able to gather information regarding the factors that you noticed as a reason for compliance or noncompliance with medications that are prescribed for patients with these conditions.I will be leading today’s discussion. My most important role is to ask questions, keep to the timeframe, and make sure that you all have the chance to share your thoughts and viewpoints. My colleague, _________ will help me with managing the discussion, and will also be taking notes. ___________ will be handling logistics(microphones and making sure you receive your reimbursements. In addition we will be audio recording the session to ensure that we don’t miss any of your answers. The discussion session today will take about1-11/2 hours total, including a break."

1. **Purpose of the focus group session**

"As I mentioned earlier, we are planning to conduct a study for developing a validated tool to measure medication adherence for selected NCDs in the Indian population .We know that you all come across many patients with these conditions . Some may be very much compliant with the drud therapy prescribed for them and some are not. The purpose of this focus group is to learn more about why some people are not adhering to the prescribed medications and some follow the exactly what is prescribed. We have asked you here to talk about your opinion and viewpoints, including reasons for their medication adherence or no adherence."

1. **Confidentiality**

"All the information we collect here today is confidential. We will use the information you provide, but we will not identify any of you in anything we do related to this meeting. For example, we will not use your name, address, or any other identifying information in reports or other materials related to this focus group."

1. **Consent forms and demographics questionnaire**

"Before we begin the discussion, I would like you to review and sign the consent form given to you when you came in. The consent form will be our record that you agreed to participate in the focus group, you agree to the recording, and you understand that we will keep information confidential. We need a signed consent form from each participant. The consent form also says that everyone in the room should respect confdentiality. If you know each other, we ask that you not talk about specifc individuals and the information they shared. It is fine to talk about the discussion, but not to identify who said what. Are there any questions about the consent form?

We would also like to collect the questionnaire that we asked you to complete when you arrived. The questionnaire will give us some information about your background. We will combine the information from all participants and use it to describe the whole group. We will never use any identifying information about you in our reports. Are there any questions about the questionnaire? Please pass your signed consent form and completed questionnaire to ________." [Note taker: Collect signed consent forms and completed questionnaires]

1. **Reimbursement/Incentive**

Describe travel reimbursement and incentive arrangements; indicate that everyone will receive them at the end of the session.

1. **Focus Group Guidelines**

"Let me begin our discussion by reviewing a few things about the focus group. We will be focusing on some specifc topics. We are interested in what everyone has to say about them. If someone throws out an idea that you want to expand on, or if you have a diferent point of view, please speak up. Sometimes I may have to interrupt the discussion to bring us back to the topic or to move on to another question or topic, to make sure that we cover everything on our agenda.

***We will follow several practical guidelines during this session:***

• We want everyone to express your opinions about the discussion topics. We are interested in diferent points of view. There are no right or wrong answers, and we are not here to resolve any issues you may bring up or to reach agreement. We just want to understand your views.

• Give us only your frst name or a nickname. No one needs identifying information about you. If you know each other, we ask that you agree to keep information confdential – if you discuss the things people said here, do not identify the people who said them.

• Feel free to agree or disagree with what other people say, while respecting their views.

• Please do not hold side conversations. We want to be able to hear from everyone, and to be able to hear what everyone says.

• Please wait to be recognized by the moderator before speaking.

• Sometimes we will go around the table to share views on a topic. You can always “pass” if you prefer not to comment on that particular topic.

• Because we are also audio-recording the session, it would really help us if you could speak up.

Do you have any questions so far?"

1. **Focus Group Discussion [Note taker: Note start time and number of participants]**

"***Participant introductions:*** Now, let’s go around the room and have each of you introduce yourselves; give your frst name, and tell us how long you have been working in NCD clinic, your current position and on an average, how many patients you usually handle a day.

Now let’s talk more about your perspectives about medication adherence among patients with NCDs mentioned.

1. **What is the general profile of patients who attend NCD clinic/receive your care?** (**Probes:** *what is their demographic profile? I mean gender, education, socioeconomic status, occupational status etc. Do they have multiple health problems or present with single disease? What is the recent trends in their prescription….whether they take one drug or mulitiple drugs for a disease? What is their practice about following a system of medicine? Do they adhere only to allopathic medicine or adopt CAM or other systems of medicine?)*
2. **What is the medication adherence behavior of patients who come across your practice ?** **(Probes:** *Could you please tell me about your observation about their medication taking pattern? In your viewpoint how important do they consider their medication in managing their health ? Are they concerned about their medication regimen? Do they discuss their doubts and concerns with you? If yes, what are the common doubts or concerns they ask to you? How do you satisfy their learning needs?* )
3. **What is your perspective about the influence of personal factors in medication compliance behavior among patients? (Probes*:*** *In your viewpoint how much they are aware about the medication/s that they take ? Do you think the gender, education or marital status influence their adherence to medication regimen? if so, what make you think so?)*
4. **Do you think that the disease condition, medication or health care personnel like you or the hospital setting affects their medication adherence behavior***? If yes, could you please explain with some examples?*
5. **Have you ever felt that cultural or religious believes and customs influence medication compliance behavior among patients? (Probes*:*** *if so, what make you to think so? Could you please make much more clear with few examples?*)
6. **Do you think that the family system has a role in medication adherence among patients? (Probes*:*** *if so, what make you think so? What you want to comment on the family support the patients receive to adhere to their medication regimen?*)
7. **In your point of view do the social and economic factors determine patients’ adherence or non adherence to their prescribed medications? (Probes*:*** *if so, what make you to think so? Could you please make much more clear with few examples? I mean the influence of job ,financial status or access to travel and pharmacy and the like*)

About halfway through the time period, give people a 10-minute break, to go to the washroom and to have some refreshments.

1. **If I ask you what is the single most important reason you personally feel for patients non adhence to their medication therapy?** **[Probes:** What could be done to keep people in line with the medications therapy?Something like DOTS or peer support from patients having similar conditions?
2. Is there anything else we should understand in order to prepare a tool to measure patients adherence to medications for NCDs?

**Closing Remarks**

"Thank you very much for participating in this focus group. The information you have provided has been very helpful. It will be used to prepare the tool mentined earlier.Are there any questions that I can answer before we end the session? Thank you again for your help. We really, really appreciate your time and your contribution. ___________ will help you pick up your transportation reimbursement before you leave."

******************************
