# Supplementary material for: Development and Validation of a Novel Tool to Measure Medication Adherence for Noncommunicable Diseases in India: Protocol for an Exploratory Sequential Mixed Methods Multicentric Study
Source: JMIR Res Protoc. 2024 Dec 3;13:e60805. doi: 10.2196/60805 (PMC11653035; doi:10.2196/60805)
Supplement: Multimedia Appendix 2 [file resprot_v13i1e60805_app2.pdf]

IDI SCHEDULE FOR PATIENTS WITH NON COMMUNICABLE DISEASES

<table>
<tr><td>Date:</td><td>Interviewer:</td><td>Interviewee:

Gender:</td><td>Start Time:</td><td>End Time:</td></tr>
<tr><td>Introduction</td><td colspan="4">“I want to thank you for taking the time to meet with me today. My name is ______________________________ and this interview is being conducted to get your input for an ICMR Project intended to develop a tool to assess the medication adherence among patients with T2 DM on OHA, hypertension, CAD or COPD/Bronchial Asthma. During this interview I would like to explore your viewpoints and experience about the reason for compliance or noncompliance with medications that are prescribed for your disease condition.”
"If it is okay with you, I will be tape recording our conversation. The purpose of this is so that I can get all the details but at the same time be able to carry on an attentive conversation with you. Although I will be taking some notes during the session, I can’t possibly write fast enough to get it all down. Because we’re on tape, please be sure to speak up so that we don’t miss your comments. All responses will be kept confidential. This means that your interview responses will only be shared with research team members and we will ensure that any information we include in our report does not identify you as the respondent. Remember, you don’t have to talk about anything you don’t want to and you may end the interview at any time. The interview today will take about one hour total, including a break. Are there any questions about what I have just explained? "
“If you agree to this interview and the tape recording, please sign this consent form."</td></tr>
<tr><td>Background?</td><td colspan="4">1.Now, let' me ask you some questions that help me to understand your personal and social background :
a) What is your age ?
b) What is your educational qualification?
c) What do you do ?....your work
d) To which economic class you belong to? I mean APL or BPL?
e) How long have you been affected by your illness?</td></tr>
</table>

	f) Do you have multiple health problems or a single disease?<br>g) Whether you take one drug or multiple drugs for your disease?<br>h) Do you adhere only to allopathic medicine or adopt other systems of medicine?
Understanding about the concept medication adherence	**2) How would you describe the term "medication adherence" in your own words?**<br>• How important do you believe medication adherence is to managing your condition?
Personal Experience and Practices	**3) Can you walk me through your daily routine for taking your medications?**<br>• Have you ever missed taking your medication? If so, what were the circumstances?<br>• Have you ever missed taking your medication according to prescribed instruction? I mean missed taking drugs on time or at correct dosage or duration…. If so, what were the circumstances?<br>• Have you ever missed/delayed your follow up appointment? If so, explain more about it?
Facilitators to Adherence	• **What helps you remember to take your medications as prescribed?**<br>• Are there specific tools or strategies you use to help with medication adherence (e.g., pillboxes, alarms)?<br>• Do you think having a support system will help you in managing your medication? I mean… family, friends or healthcare providers<br>• If I ask you the single most important reason /factor that help or remind you to take your medication as prescribed, what would it be ?<br>• Do you think knowing about why you are taking each of your medicines will help you adhere more?
Barriers to Adherence	• **What challenges or difficulties do you face in taking your medications as prescribed?**<br>• Have you ever experienced side effects from your medication? Do you think it affected your medication taking behavior<br>• Are there any financial challenges related to obtaining or affording your medications?<br>• How do your daily activities or lifestyle affect your ability to adhere to your medication schedule? I mean something like your food habits, work, travel, alcohol/smoking or such substance use….<br>• Have you ever felt that the behavior of your colleagues/friends/family members negatively influenced your medication taking behavior?<br>• Do you feel any other factors that cause difficulties for you to take your medications as prescribed?<br>• Additional probes: Fear of side effects/medication regimen complexity/shape/size?
Healthcare System and	**6)How would you describe your relationship with your healthcare provider?**<br>• Do you feel that you receive enough information and support from your healthcare provider regarding your medication?

Provider Interaction	• Have you ever had difficulties in getting your prescriptions filled or refilled? If yes, what may be the reason for not refilling the medication in time? I mean……issues with accessibility, cost or support…. something like that<br>• Have you ever had difficulties in getting appointment with your doctor?<br>• Do you think that the level of trust in your doctor influence your medication taking behavior?<br>• Do you adhere only to allopathic medicine or adopt other systems of medicine? If so,why do you adopt alternative system?
Emotional and Psychological Factors	**7) How does your emotional or mental state affect your medication-taking behavior?**<br>Do you feel frustrated/fed up with your treatment
Beliefs and Perceptions	**8) What are your thoughts on the long-term use of medication for managing your condition?**<br>**9)Are there any cultural or personal/religious beliefs that influence how you take your medication? Eg: fasting or religious rituals etc.**
Suggestions for Improvements	**10) What do you think could be done to help you better adhere to your medication regimen in your day to day life?**<br>• What advice would you give to other patients with similar conditions to help them manage their medication adherence in their day to day life?
Reflection and Conclusion	**11) Looking back, is there anything you would have done differently regarding your medication adherence?**<br>• Is there anything else you would like to share about your experience with medication adherence?
