# Supplementary material for: Development and Validation of a Novel Tool to Measure Medication Adherence for Noncommunicable Diseases in India: Protocol for an Exploratory Sequential Mixed Methods Multicentric Study
Source: JMIR Res Protoc. 2024 Dec 3;13:e60805. doi: 10.2196/60805 (PMC11653035; doi:10.2196/60805)
Supplement: Multimedia Appendix 3 [file resprot_v13i1e60805_app3.pdf]

IDI SCHEDULE FOR CAREGIVERS OF PATIENTS WITH NON COMMUNICABLE DISEASES

**Date:**	**Interviewer:**	**Interviewee:**<br><br>**Gender:**	**Start Time:**	**End Time:**
Introduction	“I want to thank you for taking the time to meet with me today. My name is ______________________________ and this interview is being conducted to get your input for an ICMR Project intended to develop a tool to assess the medication adherence among patients with T2 DM on OHA, hypertension, CAD or COPD/Bronchial Asthma. During this interview I would like to explore your viewpoints and experience about the reason for compliance or noncompliance with medications that are prescribed for these disease condition.”<br><br>"If it is okay with you, I will be tape recording our conversation. The purpose of this is so that I can get all the details but at the same time be able to carry on an attentive conversation with you. Although I will be taking some notes during the session, I can’t possibly write fast enough to get it all down. Because we’re on tape, please be sure to speak up so that we don’t miss your comments. All responses will be kept confidential. This means that your interview responses will only be shared with research team members and we will ensure that any information we include in our report does not identify you as the respondent. Remember, you don’t have to talk about anything you don’t want to and you may end the interview at any time. The interview today will take about one hour total, including a break. Are there any questions about what I have just explained? "<br><br>“If you agree to this interview and the tape recording, please sign this consent form."			

Background?	**1.Now, let' me ask you some questions that help me to understand your personal and social background :**<br><br>a) What is your age?<br>b) What is your educational qualification?<br>c) What do you do? your work<br>d) To which economic class you belong to? I mean APL or BPL?<br>e) How long have been the patient under your care diagnosed with illness?<br>f) What is your relationship with the NCD patient?<br>g) Do you provide care for patients with multiple health problems or a single disease?<br>h) Whether they take one drug or multiple drugs for your disease?<br>i) Do they adhere only to allopathic medicine or adopt other systems of medicine?
Understanding about the concept medication adherence	**2) How would you describe the term "medication adherence" in your own words?**<br><br>• How important do you believe medication adherence is to managing the NCDs?
Personal Experience and Practices	**3) Can you walk me through their daily routine for taking medications?**<br><br>• Have you ever noticed them missed taking their medication? If so, what were the circumstances?<br>• Have they ever missed taking medication according to prescribed instruction? I mean missed taking drugs on time or at correct dosage or duration…. If so, what were the circumstances?<br>• Have they ever missed/delayed follow up appointment? If so, explain more about it?
Facilitators to Adherence	• **What do you think help them remember to take their medications as prescribed?**<br>• Are there specific tools or strategies they use to help with medication adherence (e.g., pillboxes, alarms)?<br>• Do you think having a support system will help you in managing your medication? I mean… family, friends or healthcare providers<br>• If I ask you the single most reason /factor that help or remind them to take their medication as prescribed, what it would be ?<br>• Do you think knowing about why they are taking their medicines will help them adhere more?
Barriers to Adherence	• **What challenges or difficulties do you think they face in taking their medications as prescribed?**<br>• Have they ever experienced side effects from medication? Do you think it affected their medication taking behavior

	• Are there any financial challenges related to obtaining or affording their medications?<br>• Do you think their daily activities or lifestyle affect their ability to adhere to medication schedule? I mean something like their food habits, work, travel, alcohol/smoking or such substance use….<br>• Have you ever felt that the behavior of colleagues/friends/family members negatively influenced medication taking behavior?<br>• Do you feel any other factors that cause difficulties to take medications as prescribed?<br>• Additional probes: Fear of side effects/medication regimen complexity/shape/size?
Healthcare System and Provider Interaction	**6)How would you describe the relationship with your family member with healthcare provider?**<br><br>• Do you feel that you receive enough information and support from healthcare provider regarding medication?<br>• Have you ever had difficulties in getting prescriptions filled or refilled? If yes, what may be the reason for not refilling the medication in time? I mean……issues with accessibility, cost or support…. something like that<br>• Have you ever had difficulties in getting appointment with doctor?<br>• Do you think that the level trust in the treating doctor influence their medication taking behavior?<br>• Do they adhere only to allopathic medicine or adopt other systems of medicine? If so,why do you think they adopted alternative system?
Emotional and Psychological Factors	**7) How do you think emotional or mental state affect the medication-taking behavior of your care reciever?**
Beliefs and Perceptions	**8) What are your thoughts on the long-term use of medication for managing this condition?**<br><br>**9)Are there any cultural or personal/religious beliefs that influence taking the medication? Eg: fasting or religious rituals etc.**
Suggestions for Improvements	**10) What do you think could be done to help your care reciever better adhere to medication regimen in their day to day life?**

Reflection and Conclusion	11) Looking back, is there anything else you would like to share about your experience with medication adherence?
