# Supplementary material for: Development and Validation of a Novel Tool to Measure Medication Adherence for Noncommunicable Diseases in India: Protocol for an Exploratory Sequential Mixed Methods Multicentric Study
Source: JMIR Res Protoc. 2024 Dec 3;13:e60805. doi: 10.2196/60805 (PMC11653035; doi:10.2196/60805)
Supplement: Multimedia Appendix 4 [file resprot_v13i1e60805_app4.docx]

**PARTICIPANT INFORMATION SHEET – QUALITATIVE STUDY**

**Aims of the study**

Currently, we do not have a validated medication adherence tool contextualized to the Indian setting that could be utilized for the measurement of adherence across various chronic diseases.

We, with funding from the Indian Council of Medical Research (ICMR) envisage developing a novel indigenous medication adherence tool for select Non communicable diseases (NCDs) namely Type II Diabetes mellitus, Hypertension, Coronary artery disease (CAD), Chronic obstructive pulmonary disease (COPD) / Bronchial asthma, which is ethnically and culturally appropriate. The various constructs of the tool would be developed after exploration of expert opinions and patients' perspectives on adherence behavior. To ensure that the contents are relevant to the local population, the tool would be validated across four geographical zones of India

**What is your role?**

You can be a part of this study after providing written consent. The study team will collect basic socio-demographic details from you. Our study team will contact you to interview and enquire your perceptions regarding medication adherence to the above-mentioned diseases.

**What will you gain from this study?**

There are no financial gains. By being a part of this study, you are providing valuable data which can help us develop a novel medication adherence tool, and validate it for measuring adherence among patients with chronic diseases. This would in turn help treating physicians and other health care workers in improving interventions for medication adherence.

**Are there any risks for getting involved in this study?**

There are no potential risks for getting involved in this study. No lab investigation or medication would be given.

**Will the information which you provide be kept confidential?**

The information you provide, including your name will be kept confidential. Data would be used for research purposes and publication only after removal of personal identifiers.

**Whom to contact in case of any enquiry?**

Principal Investigator-Dr Joe Thomas, Professor, Department of Community Medicine, Jubilee Mission Medical College & Research Institute, Thrissur. Ph: 8593841000

**CONSENT FORM - QUALITATIVE STUDY**

**Title of the study**: **Development and validation of a novel tool to measure medication adherence for select NCDs in the Indian population - An Exploratory sequential mixed method multicentric study**

Name of the Participant: ........................................

(i) I confirm that I have read and understood the information sheet dated ____________ for the above study and have had the opportunity to ask questions. [ ]

(ii) I understand that my participation in the study is voluntary and that I am free to withdraw at any time, without giving any reason, without my medical care or legal rights being affected. [ ]

(iii) I understand that the Ethics Committee and the regulatory authorities will not need my permission to look at the transcripts both in respect of the current study and any further research that may be conducted in relation to it, even if I withdraw from the study. I agree to this access. However, I understand that my identity will not be revealed in any information released to third parties or published. [ ]

(iv) I agree not to restrict the use of any data or results that arise from this study provided such a use is only for scientific purpose(s). [ ]

(v) I agree to take part in the above study. [ ]

Signatory’s Name: _________________________________ Signature:

Date: _____/_____/______

Statement by researcher:

I have read out the information sheet to the potential participant, and to the best of my ability made sure that the person understands that the study procedures and risks. I confirm that the participant was given an opportunity to ask questions about the study, and all the questions asked have been answered correctly and to the best of my ability. I confirm that the individual has consented freely and voluntarily. A copy of this consent form has been provided to the participant.

Researcher’s Name:

**Principal Investigator contact details**:

Dr Joe Thomas

Professor, Department of Community Medicine,

Jubilee Mission Medical College & research Institute, Thrissur, Kerala – 680005

Mobile: 8593841000

Email: covidjmmcri@gmail.com

**PARTICIPANT INFORMATION SHEET – CROSS SECTIONAL STUDY**

**Aims of the study**

Currently, we do not have a validated medication adherence tool contextualized to the Indian setting that could be utilized for the measurement of adherence across various chronic diseases.

We, with funding from the Indian Council of Medical Research (ICMR) envisage developing a novel indigenous medication adherence tool for select Non communicable diseases (NCDs) namely Type II Diabetes mellitus, Hypertension, Coronary artery disease (CAD), Chronic obstructive pulmonary disease (COPD) / Bronchial asthma, which is ethnically and culturally appropriate. The various constructs of the tool would be developed after exploration of expert opinions and patients' perspectives on adherence behaviour. To ensure that the contents are relevant to the local population, the tool would be validated across four geographical zones of India

**What is your role?**

You can be a part of this study after providing written consent. The study team will collect basic socio-demographic details, details of your disease condition, current medications and questions regarding medication adherence from the tool that was developed. The health worker will visit you again after 30 days and verify your adherence behavior.

**What will you gain from this study?**

There are no financial gains. By being a part of this study, you are providing valuable data which can help us develop a novel medication adherence tool, and validate it for measuring adherence among patients with chronic diseases. This would in turn help treating physicians and other health care workers in improving interventions for medication adherence.

**Are there any risks for getting involved in this study?**

There are no potential risks for getting involved in this study. No lab investigation or medication would be given.

**Will the information which you provide be kept confidential?**

The information you provide, including your name will be kept confidential. Data would be used for research purposes and publication only after removal of personal identifiers.

**Whom to contact in case of any enquiry?**

Principal Investigator-Dr Joe Thomas, Professor, Department of Community Medicine, Jubilee Mission Medical College & Research Institute, Thrissur. Ph: 8593841000

**CONSENT FORM - CROSS SECTIONAL STUDY**

Title of the study: **Development and validation of a novel tool to measure medication adherence for select NCDs in the Indian population - An Exploratory sequential mixed method multicentric study**

Name of the Participant: ........................................

(i) I confirm that I have read and understood the information sheet dated ____________ for the above study and have had the opportunity to ask questions. [ ]

(ii) I understand that my participation in the study is voluntary and that I am free to withdraw at any time, without giving any reason, without my medical care or legal rights being affected. [ ]

(iii) I understand that the Ethics Committee and the regulatory authorities will not need my permission to look at the transcripts both in respect of the current study and any further research that may be conducted in relation to it, even if I withdraw from the study. I agree to this access. However, I understand that my identity will not be revealed in any information released to third parties or published. [ ]

(iv) I agree not to restrict the use of any data or results that arise from this study provided such a use is only for scientific purpose(s). [ ]

(v) I agree to take part in the above study. [ ]

Signatory’s Name: _________________________________ Signature:

Date: _____/_____/______

Statement by researcher:

I have read out the information sheet to the potential participant, and to the best of my ability made sure that the person understands that the study procedures and risks. I confirm that the participant was given an opportunity to ask questions about the study, and all the questions asked have been answered correctly and to the best of my ability. I confirm that the individual has consented freely and voluntarily. A copy of this consent form has been provided to the participant.

Researcher’s Name:

**Principal Investigator contact details**:

Dr Joe Thomas

Professor, Department of Community Medicine, JMMC & RI, Thrissur, Kerala -680005

Mobile: 8593841000

Email: covidjmmcri@gmail.com
