# Supplementary material for: Development and Validation of a Novel Tool to Measure Medication Adherence for Noncommunicable Diseases in India: Protocol for an Exploratory Sequential Mixed Methods Multicentric Study
Source: JMIR Res Protoc. 2024 Dec 3;13:e60805. doi: 10.2196/60805 (PMC11653035; doi:10.2196/60805)
Supplement: Multimedia Appendix 5 [file resprot_v13i1e60805_app5.docx]

# To, Date: 02/07/2024

**The Editor**

# JMIR Research Protocols

**Subject: Confirmation of Proposal Review and Protocol Development by ICMR as funding agency for the project.**

Dear Editor,

We are pleased to inform you that Dr Joe Thomas’s project protocol titled ‘***Development and Validation of Novel Tool to Measure Medication Adherence for Select NCDs in the Indian Population: Protocol for an Exploratory Sequential Mixed Method Multi-centric Study - Proposal id: IIRP-2023-0000003,(2023-20064)’*** has successfully undergone protocol development and thorough reviews facilitated by the **ICMR Central Coordinating Unit (CCU)**

# - National Task Force (NTF) on Safe and Rational use of Medicines (SRUM).

**Protocol Development Workshop:** As mentioned in the earlier letter dated 26 June 2024, the project was conceived at ICMR using a research priority setting exercise and individual studies were developed into full protocols at a 2-day workshop at ICMR.

**Review Process by the Technical Advisory Group (TAG):** Additionally, the proposal has been reviewed by the ICMR Technical Advisory Group (TAG) which comprises experts from diverse fields including research methodology, biostatistics, pharmacology, and clinical research, ensuring that it meets the highest standards of scientific rigor and relevance.

Following these comprehensive reviews and development sessions, the proposal has been refined and deemed suitable for funding. We believe that the rigorous evaluation it has undergone signifies the quality and significance of the work.

The comments from the Technical Advisory Group (TAG) regarding the proposal are annexed to this letter as requested by the journal.

Best regards,

Indian Council of Medical Research Headquarters.

| **Proposal which needs minor revision Slot#10 Dr. Joe Thomas**  **Development and validation of a novel tool to measure medication adherence for select NCDs in the Indian population - An Exploratory sequential mixed method multicentric study** | | |
| --- | --- | --- |
| **General comments from TAG** | | **Details of revision made to proposal/ comments/ response by applicants** |
| 1 | To include appropriate subject experts with necessary expertise in the project team to strengthen and guide the project implementation. Many of the applicants seem to lack necessary expertise required for developing and implementing the assigned studies. This is expected when non-medical interventions are planned, however appropriate experts may be identified with the help of ICMR to be included in the revised proposal. All the proposals on tool development and validation need to work much more on validity assessment. | Dr Ravi Prasad Varma has been included as our consultant. He is an expert in tool development and has published many papers in indexed journals. He is a renowned resource person and has conducted many workshops on tool validation, item reduction analysis and qualitative studies. |
| 2 | SRUM Low priority activities (including prescription research and prescriber skill course) will have to be accounted for in the activities of the site. Budget in revised proposal may include accounting for necessary resources to complete the low priority activities. Justification for the budget can mention responsibilities for low priority activities. | 1 Data entry operator has been accounted in the budget proposal for the project and low priority activities |
| 3 | The Task Force will have to eventually assimilate all the data gathered across the various projects to assess the overall impact. For this purpose, various indicators have been developed. The applicants may include as many relevant health systems output indicators in the proposals as possible so that after completion, the impact of various projects can be cumulated. This will include the following, if relevant   a. Number of deaths prevented b. Number of ICU admissions averted c. Number of readmissions averted d. Impact on morbidity, mortality etc. e. Unnecessary medicines discontinued | Not applicable |

| 4 | Need more details regarding the significance of the assigned project topic in Global and Indian context. The applicants could not adequately justify the need for an adherence tool despite of the availability of many such tools, for which a quick review of current global and Indian scenarios may be presented at the outset in the revised proposal. This could also be included as a deliverable to conduct and publish a review of similar tools.  **Response:**  India is undergoing an epidemiological health transition with an increasing burden of non-communicable diseases (NCDs). NCDs contribute to around 5.87 million deaths which account for 60 % of all deaths in India which is two-thirds of the total deaths due to NCDs in the South-East Asia Region (SEAR) of WHO (1).  In developed countries, only 50% of the patients treated for chronic diseases adhere to the prescribed treatment and the problem in developing countries is greater (1). The key contributor to the increasing burden of morbidity and mortality due to NCDs is poor control status of the patients, which is in turn due to poor medication adherence (2). Diabetes, Hypertension and bronchial asthma rank among the highest diseases reporting medication non-adherence (3, 4). Improving medication adherence essentially requires assessment using a valid and reliable adherence scale, considering the various dimensions of adherence. Objective measures include pill counts, electronic monitoring, secondary database analysis and biochemical measures which are often impractical and resource intensive. Subjective measures like self-reports and health care professional assessments rely on data which could be influenced by social desirability bias, recall bias and might not capture the complex cultural, and socioeconomic factors. Nevertheless, it has benefits of being cost effective, non-intrusive, user friendly, easy to administer and able to capture patients concerns and thereby individualize adherence interventions.  Globally, the psychometric properties of different medication adherence scales have demonstrated heterogeneous results when applied in different settings and disease conditions. Studies by Sakthong P et al, and Zongo A et al, reported that the Morisky Medication Adherence Scale (MMAS) demonstrated sensitivity as low as 51% and low reliability values in T2DM patients (5, 6). The Medication Adherence Report Scale (MARS 5) was found to be less sensitive in measuring medication adherence to asthma and other chronic disease conditions (7,8). In India, non-adherence to medication varies from 18.7% to 74%, (2, 9, 10, 11) which is assessed using scales validated in the Western population. Currently, we do not have a validated medication adherence tool contextualized to the Indian setting. The phrasing of questions in scales validated elsewhere and its interpretations may vary when applied in the Indian patients. Moreover, these scales have not accounted for the unique cultural, social, and economic factors influencing medication adherence in India. This could result in inaccurate reports of adherence behavior. Besides these scales often require licensing charges. Furthermore, only limited studies have looked at the psychometric properties of adherence scales such as MMAS, MARS, BMQ, ARMS in the Indian context (12, 13). We envisage developing a novel indigenous medication adherence tool for select NCDs, which is ethnically, culturally and linguistically appropriate. The various constructs of the tool would be developed after exploration of expert opinions and patients' perspectives on adherence behavior. To ensure that the contents are relevant to the local population, the tool would be linguistically validated across different geographical zones of India. Unlike other medication adherence scales which have been validated in hospital settings, where patients are likely to be more adherent, we propose to validate our tool in the community settings which would give a more accurate real-world perspective of adherence. This tool, which is envisaged to be developed as two versions, could be used across chronic disease conditions both in routine clinical practice (short version) and research settings (long version). This can help the policy makers understand and identify the reasons and consequently adopt tailored policies to improve adherence. Thus, the present study aims to develop a medication adherence tool and evaluate its |
| --- | --- |

|  | psychometric properties to measure adherence for select NCDs in the Indian population. | |
| --- | --- | --- |
| 5 | Break up of various activities in Gantt chart to allow for monitoring of progress may be mentioned in revised proposal along with clear annual deliverables, which could include achievement of study landmarks, publications, details of meetings/ capacity building activities attended etc.   | |
| 6 | All proposal submissions to utilize the template for clinical trial which was shared by ICMR. Non-trial studies can use the same template with suitable modifications. All documents to be included as single PDF, preferably with hyperlinks to relevant sections. | Complied with the suggestion and modified |
| **Proposal specific comments** | | |
| 1 | What are your justifications for developing a new tool for measuring medication adherence while many other tools are there to measure the same. Mention the advantages of your intervention over other existing tools in detail.  **Response:**  In the low-resource settings of the developing world, the existing medication adherence scales need to be cautiously applied for the following reasons: First, most of these questionnaire scales which were originally validated in the Western world should be assessed for cross-cultural equivalence taking into account the diverse linguistic, socioeconomic, cultural, extremes of literacy prevalent in Indian settings. The phrasing of questions in scales validated elsewhere and its interpretations may vary when applied in the Indian patients. This would result in mixed findings on accuracy, reliability and validity in patients with various chronic disease | |

|  | conditions. Furthermore, India has mixed health care systems, where people’s access to and use of medicines are affected by their socioeconomic background. The out-of-pocket expenditure is an important area of health economics especially in developing countries where most patients pay direct medical costs. The existing tools lack this important domain in measurement of adherence. Most adherence questionnaires should preferably be self-administered, but in patients with low-literacy, they need to be verbally administered by an assessor that can influence their reliability and validity while introducing the risk of bias.  Questionnaires with more items take proportionally more time to fill which in overcrowded clinic settings is a drawback. Only limited studies have looked at the psychometric properties of adherence scales such as MMAS, MARS in the Indian context. Besides these scales often require licensing charges. We envisage developing a novel indigenous medication adherence tool for select NCDs, which is ethnically, culturally and linguistically appropriate. The various constructs of the tool would be developed after exploration of expert opinions and patients’ perspectives on adherence behavior. To ensure that the contents are relevant to the local population, the tool would be linguistically validated across different geographical zones of India. Unlike other medication adherence scales which have been validated in hospital settings, where patients are likely to be more adherent, we propose to validate our tool in the community settings which would give a more accurate real-world perspective of adherence. This tool, which is envisaged to be developed as two versions, could be used across chronic disease conditions both in routine clinical practice (version) and research settings (long version). This can help the policy makers understand and identify the reasons and consequently adopt tailored policies to improve adherence. |
| --- | --- |
| 2 | What are the differences of your intervention with the existing tools for measuring medication adherence? Please describe in detail.  **Response:**   1. The new tool would be developed after an initial deductive and inductive process. Items would be generated by deductive process, which would further be validated by the inductive process, where exploratory research methodologies would be used. 2. Cognitive piloting would be done involving patients with disease conditions under study. Based on patients’ comprehension of questions changes will be made to make the tool acceptable & parsimonious. 3. The proposed tool would include dimensions such as: patient behavior, co-morbidity, pill burden, self- efficacy, cost factors and components of family dynamics, which is valid in Indian settings 4. The reasons for non-adherence (intentional and unintentional) for medication would be assessed 5. The tool can be used in patients with all levels of literacy. For enabling responses from patients with low literacy, some of the items would have pictograms 6. The development and validation process involves robust mathematical algorithms. 7. We propose to estimate convergent validity against objective measures such as direct pill count and MEMS 8. Our tool would be validated across four geographical zones of India to account for the diverse socio- economic, cultural and linguistic differences (Hindi, Marathi, Assamese and Malayalam) 9. Validation in community settings as against hospital settings done in other tools would pick up the real- |

|  | world evidence on adherence  10. The 2-3 best fit global questions, which would be selected after considering the content validity indices, consensus by experts and communality, would be a simple, and rapid method of assessment of medication adherence at the point of care/clinical settings. |
| --- | --- |
| 3 | Suggestion to do more scoping review to find out the gaps in the existing tools. What do you mean by a tool specific to Indian context while other tools can adequately measure it? If the present tools have been used in India, please describe the limitations with evidence.  **Response:**  The most commonly used adherence scales are the Morisky Medication Adherence Questionnaire (MMAS 4, MMAS 8), Self-efficacy for Appropriate Medication Use Scale (SEAMS), Beliefs about Medicines Questionnaire (BMQ), Medication Adherence Rating Scale (MARS, MARS 5), Hill Bone compliance scale, Adherence to Refills and Medications Scale (ARMS) and General Medication Adherence Scale (GMAS) (14 - 22). However, these adherence scales have their own strengths and limitations. The majority of these scales have been developed and validated in settings, which are strikingly in contrast to the Indian scenario. Measuring adherence using scales validated elsewhere, would not take into account the diverse linguistic, socioeconomic, cultural, extremes of literacy prevalent in Indian settings. This would result in mixed findings on accuracy, reliability and validity in patients with various chronic disease conditions. Furthermore, India has mixed health care systems, where people’s access to and use of medicines are affected by their socioeconomic, cultural, and religious backgrounds. The out-of-pocket expenditure is an important area of health economics especially in developing countries where most patients pay direct medical costs (23, 24). Thus, the existing tools lack this important domain in measurement of adherence.  The best known and most widely used is medication adherence questionnaire (MAQ) by Morisky et al (14, 15). The Morisky Scale has been developed from the 4-item MMAS-4 to the 8-item MMAS-8. The scale is short and quick to administer. The initial version of the scale validated in hypertensive patients had a sensitivity of 81%, a specificity of 44%, and internal consistency of α = 0.61. In the MMAS-8, seven items are (yes/no) responses and the last item is a 5-point Likert response. The specificity and the sensitivity of the MMAS-8 were (53% and 93%), respectively. Barriers identified are mainly forgetfulness and adverse effects. Cost related non adherence (CRNA), self-efficacy and health care system related domain is not captured in MMAS (23, 24). In addition, there is overlap between the questions which could result in scoring ambiguity. For example: Question 5 in MMAS (Did you take all your medicines yesterday?), has answer no’, then automatically, question 1 (Do you sometimes forget to take your pills?) and 8 (How often do you have difficulty remembering to take all your medicines) would also be scored. So, this would result in a total score of three with only one answer.  Study in Thailand (5), among Type 2 DM patients, reported low sensitivity and predictive accuracy (51% and 43% respectively) with a moderate specificity of 64%. The Spanish version of the Morisky scale was found not to be suitable for measuring medication adherence in T2 DM as evidenced by low internal consistency seen in study by Martinez-Perez, P et al (25). Additionally, a study by Wang, Y et al, among T2DM patients in Singapore (26), reported that the psychometric properties of the modified MMAS 4 scale were less than satisfactory and ceiling effect was observed. They also reported that this scale may not be an adequate measure to assess |

|  | medication adherence.  The Medication Adherence Report Scale (MARS), explores beliefs and barriers to medication-taking behavior. It includes ten questions that assess adherence behavior and disease control over the past week and used for patients with chronic mental illness. The reliability of the scale was 0.75, sensitivity and specificity were not reported (18). The shorter version MARS- 5 (19) performed well on several psychometric indicators and validated in hypertension, asthma and diabetes patients. The major limitations were the scale not validated in low literacy patients and do not assess self-efficacy. A study on psychometric evaluation of the MARS in caregivers of low-income, urban, African American children with poorly controlled asthma, by Margolis R et al (7), demonstrated marginal fit in Confirmatory Factor Analysis and there was lack of associations with objective measures, hence not clinically indicated in assessment of asthma medication adherence Whereas, study by [Maria Lia Scribano](https://pubmed.ncbi.nlm.nih.gov/?term=Scribano%2BML) et al reported that, Italian translation of MARS, showed satisfactory internal consistency, test-retest, but low convergent validity (27). The assessment of medication adherence showed a difference when measured using Indian version of compliance questionnaire rheumatology (CQR) and MARS as shown by the mean cut point differences; 86.4% (CQR) Vs 74.29% (MARS) (28).  Adherence to Refills and Medication scale (ARMS)**,** a 12-item scale evaluating taking medications as prescribed and refilling medications on schedule. It had high internal consistency overall as well as in patients with inadequate literacy skills (overall reliability 0.814) and correlated well with Morisky scale (20). Cost factor and out of pocket expenditure, patient self-efficacy dimensions of medication adherence were not considered in this scale. Additionally, sensitivity, specificity of ARMS is not reported and compared to clinical outcomes. We do not find any studies that have validated ARMS in the Indian setting.  The Self-efficacy for Appropriate Medication Use scale (SEAMS) and the Brief Medication Questionnaire (BMQ) has three main question headings and multiple sub questions (16, 17). Both assess self-efficacy, barriers and are validated in patients with low literacy. However, the sensitivity and specificity of SEAMS is not estimated and it lacks the ability to rapidly estimate adherence at point of care/bedside. Hill-Bone compliance scale (21) is validated in hypertensive patients predominantly black population with a reliability of 0.65 limited in their generalizability, scoring at bed side and low literacy patients. The MMAS, BMQ, as well as ARMS are not suitable for patients for whom out-of-pocket medical expenditures are a barrier to adherence (29). In a study by Basu *et al*., among Type 2 diabetes patients in a tertiary care hospital in Delhi, the rate of medication nonadherence assessed by the MMAS-8 item and self-reported lack of medication possession during the previous 3 months differed significantly at 25.5% and 41%, respectively (30).  Most of these medication adherence scales are validated in a single disease population and time for administration varies from 5 minutes (for less than 5 items) to 20-25 minutes. Majority of these scales have compared measures of adherence against other subjective measures or clinical outcomes. Very few scales have estimated sensitivity and specificity, which is an important component of convergent validity and only a few scales such as BMQ were validated against MEMS, which is an objective measure of adherence (17). A small number of adherence scales have assigned an adherence cut off (MAQ, MMAS, BMQ) (14, 15, 17). Majority of these scales have been validated in a relatively small sample of patients, which is a limitation against generalizability. Dichotomizing adherence responses with a yes/no response did not differentiate between highly adherent and partially adherent patients or patients at different stages of the medication taking process. Some scales focus more on medication taking behavior (MMAS) whereas others seek detailed information about barriers and patient beliefs that may influence adherence (BMQ). Scales such as BMQ are not stand- alone comprehensive adherence scales, which calls for need of developing scales that provide opportunity for a |
| --- | --- |

|  | comprehensive assessment of patient’s adherence and drivers behind the adherence. In a systematic review by Forbes et al, which evaluated the strengths and limitations of the various medication adherence tools such as Shea, MMAS, ARMS and BMQ and found that, there was inaccuracy, over-estimation of adherence, complexity of scale, and the cost associated with the license agreement of MMAS were found as few limitations of the scales used.  In India, there are vast socioeconomic barriers, inequalities in access to treatment, suboptimal staffing in health-care facilities and limited supply of medication. In order for health care providers to promote medication adherence, an easy to use, reliable and valid measure of medication adherence is needed. In a Study conducted by Basu S et al in Delhi, none of the health care professionals were aware of validated scales for medication adherence. (Hence, we intend to develop a shorter version of the medication adherence tool which could be integrated into routine clinical practice based on content validity results and consensus from expert panel review. |
| --- | --- |
| 4 | Please specify the details of the PI and Co-PI expertise in developing a new tool and using relevant mathematical algorithms in your protocol.  **Response:**  The PI & the Co-PIs have experience in community-based projects, medication adherence, ADR surveillance, medication management in NCDs but not specifically in tool validation. However, our consultant Dr Ravi Prasad Varma has been involved in tool development and has published landmark papers, the references of which are given below. He is well versed with all the mathematical algorithms we have mentioned in our proposal.  He was involved in drafting our proposal. He was also a facilitator for tool validation of ICMR for capacity building sessions of SRUM applicants.  References of Tool Validation papers of Dr Ravi Prasad Varma   1) Kochuvilayil A, Varma RP. Caregiver Burden among Informal Caregivers in the Kerala Palliative Care Program: Development and Validation of the Achutha Menon Centre-Caregiver Burden Inventory. J Palliat Med. 2021 Aug;24(8):1197-1205. doi: 10.1089/jpm.2020.0477. Epub 2020 Dec 28. 2) Kaul S, Paplikar A, Varghese F, Alladi S, Sharma M, Dhaliwal RS, Goyal S, Varma RP et al, Consortium ICMR CTB. MoCA in five Indian languages: A brief screening tool to diagnose dementia and MCI in a linguistically diverse setting. Int J Geriatr Psychiatry. 2022 Aug 31;37(10). doi: 10.1002/gps.5808. Epub ahead of print. 3) Verma M, Tripathi M, Paplikar A, Narayanan J, Varma RP, et al. Validation of ICMR Neurocognitive Toolbox for Dementia in the Linguistically Diverse Context of India. Front Neurol. 2021 Oct 18; 12:661269. doi: 10.3389/fneur.2021.661269. 4) Paplikar A, Iyer GK, Varghese F, Alladi S, Pauranik A, Mekala S, Kaul S, Sharma M, Dhaliwal RS, Varma RP et al; ICMR Neuro-Cognitive Tool Box Consortium. A Screening Tool to Detect Stroke Aphasia: Adaptation of Frenchay Aphasia Screening Test (FAST) to the Indian Context. Ann Indian Acad Neurol. 2020 Sep;23(Suppl 2): S143 - |

|  | S148. doi: 10.4103/aian.AIAN_499_20. Epub 2020 Sep 25.  5) Jose DS, Varma R, Sambasivan SL, Philip L, Sandhya P, Sundaram S. Autism Spectrum Disorders: Barriers to Implementing Parent-Based Home Programs. Indian J Pediatr. 2023 Jan;90(1):79-82. doi: 10.1007/s12098 -022- 04383-x. Epub 2022 Nov 18. (Had barriers scale development) |
| --- | --- |
